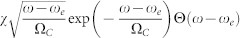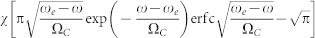# Breakdown of Bose-Einstein Distribution in Photonic Crystals

**DOI:** 10.1038/srep09423

**Published:** 2015-03-30

**Authors:** Ping-Yuan Lo, Heng-Na Xiong, Wei-Min Zhang

**Affiliations:** 1 Department of Physics and Center for Quantum Information Science, National Cheng Kung University, Tainan 70101, Taiwan, Republic of China

## Abstract

In the last two decades, considerable advances have been made in the investigation of nano-photonics in photonic crystals. Previous theoretical investigations of photon dynamics were carried out at zero temperature. Here, we investigate micro/nano cavity photonics in photonic crystals at finite temperature. Due to photonic-band-gap-induced localized long-lived photon dynamics, we discover that cavity photons in photonic crystals do not obey Bose-Einstein statistical distribution. Within the photonic band gap and in the vicinity of the band edge, cavity photons combine the long-lived non-Markovain dynamics with thermal fluctuations together to form photon states that memorize the initial cavity state information. As a result, Bose-Einstein distribution is completely broken down in these regimes, even if the thermal energy is larger or much larger than the cavity detuning energy. In this investigation, a crossover phenomenon from equilibrium to nonequilibrium steady states is also revealed.

Photonic band gap (PBG) structures in photonic crystals (PCs) together with the characteristic dispersion properties have stimulated considerable interest in the study of fundamental photonic science and also in the development of new photonic technology^1^,^2^,^3^. The most significant new features induced by the PBG are the inhibition of atom spontaneous emission and the localization of light^4^,^5^,^6^. As a result, ultrahigh quality-factor cavity has been realized on-chip with PBG structures^7^. This provides the opportunity to control and manipulate light for photonic information technology^8^. In the past two decades, quantum optics with a few-level atom placed inside PCs have been extensively explored^9^, and cavity QED with the features of atomic population trapping and atom-photon bound states in the vicinity of the photonic band edge (PBE) has been examined^10^,^11^,^12^,^13^,^14^. These features are obtained mainly at zero temperature by solving the Schrödinger equation in which the PCs contain only one single photon emitted from an atom which is initially in the excited state. On the other hand, when the number of photons increases, light propagating in PCs was understood using the classical Maxwell equations, which is also defined at zero temperature^1^,^3^. Indeed, photonic quantum dynamics, even for a pure micro/nano cavity embedded in PCs, has not yet been solved at finite temperature. Practically, understanding photonic quantum dynamics at finite temperature is important for the development of all-optical circuits incorporating cavities and PBG waveguides embedded in PCs in the microwave regime.

All-optical circuits for networks on-chips consist of micro/nano cavities and waveguides^8^. Micro/nano cavities in PCs are created by point defects, and PBG waveguides can be made with coupled defect arrays. Frequencies of cavities and waveguides can be easily tuned by changing the size and/or the shape of defects. In this article, we investigate micro/nano cavity photonics in PCs at finite temperature. Due to PBG-induced localized long-lived non-Markovian photon dynamics, we find that cavity photons in PCs do not obey Bose-Einstein statistical distribution. Within the PBG region and also in the vicinity of the PBE, cavity photons combine the nontrivial non-Markovian dissipations with thermal fluctuations together to form photon states that can memorize the initial cavity state information. As a result, Bose-Einstein statistical distribution for photons is completely broken down in these regimes, even though the photonic thermal energy is larger or much larger than the cavity detuning energy. Also, a crossover phenomenon from equilibrium to nonequilibrium steady states is revealed in low-dimensional photonic crytslas.

## Results

### Dissipation and dissipationless photon dynamics in photonic crystals

The methodology for the study of cavity photon dynamics in photonic crystals at finite temperature was developed in our previous work^15^,^16^,^17^,^18^, which is summarized in the Methods Section in the end of the article. To investigate the evolution of cavity photon states in PCs, we shall solve first the dynamics of photon dissipation and fluctuations, determined by Eqs. (19)–(20) given in Methods. Different spectral density *J*(*ω*) of the PCs, which requires the knowledge of DOS of the PCs, will provide different photon dissipation and fluctuations. In standard quantum optics, the spectral density *J*(*ω*) is usually treated as a constant in the weak-coupling limit, where the Weisskopf-Winger approximation or the Markovian master equation is valid so that the photon damping rate is time-independent (i.e. memoryless). As a result, all cavity photon modes have a finite lifetime and the cavity photon states will ultimately evolve into thermal equilibrium with the environment after decoherence takes place^19^, and photons inside the cavity must obey Bose-Einstein distribution^20^,^21^,^22^. However, this well-known result is no longer satisfied for micro/nano cavities in PCs, due to the presence of the PBG, as we will show below.

**Figure 1:**
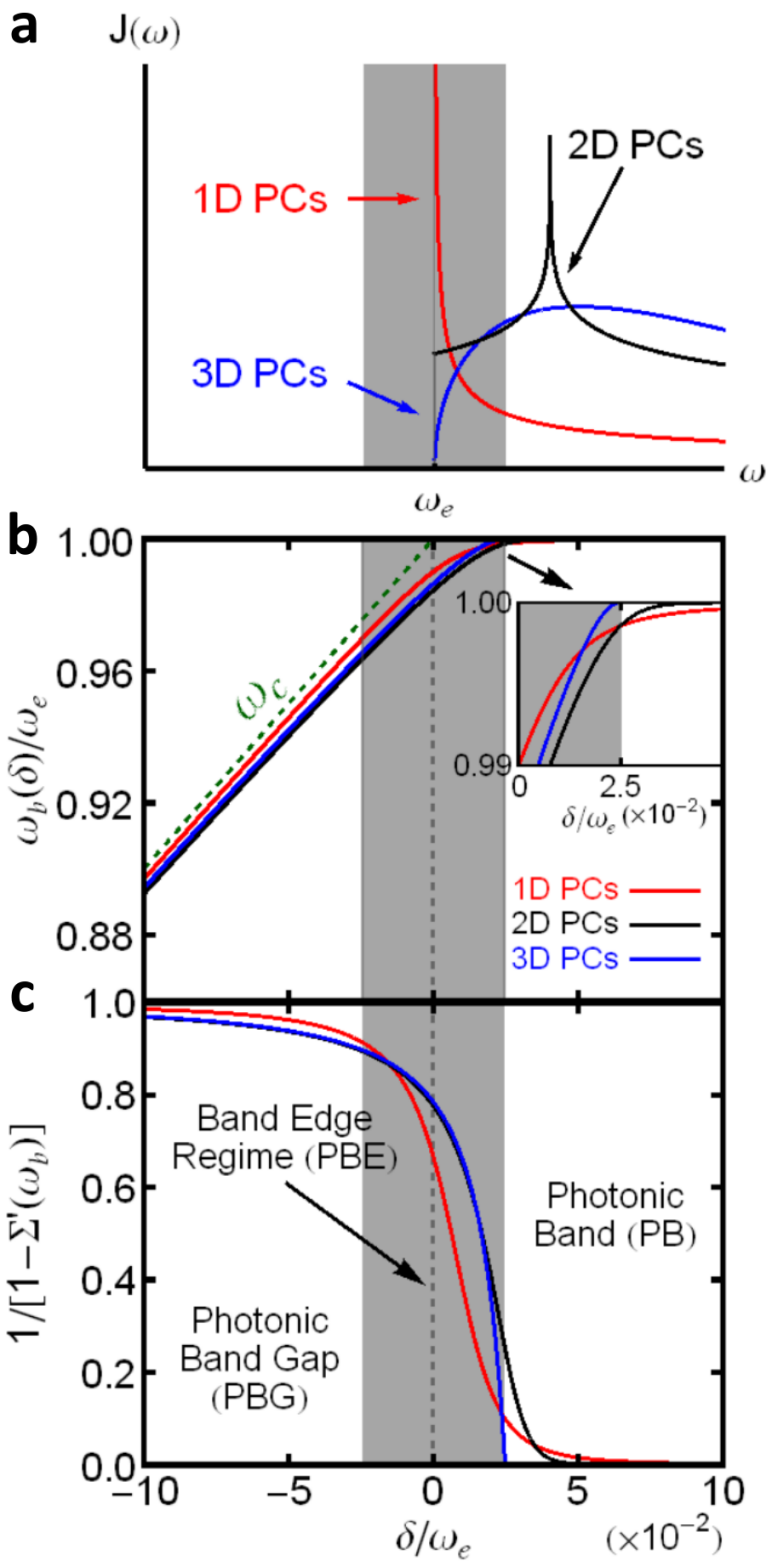
Band structures of photonic crystals and localized photon modes. (a) Spectral densities listed in Table 1 for different DOS of 1D, 2D and 3D PCs (with different colors) are plotted respectively in the vicinity of photonic band edge *ω_e_*; (b) The corresponding localized photon mode frequency *ω_b_* as a function of the detuning *δ* = *ω_c_* − *ω_e_*; and (c) The corresponding localized photon mode amplitudes, given in Eq. (2). The localized photon mode shows a crossover for 1D and 2D PCs, and a critical transition for 3D PCs when the cavity frequency *ω_c_* changes from the PBG region to the PB region. The parameters given in Table 1 take as follows: the coupling strengths for 1D, 2D and 3D PCs are *C*^2/3^ = 0.01*ω_e_*, *η* = 0.001*ω_e_* and *χ* = 0.014*ω_e_*, respectively. Experimentally, the typical photonic band edge frequency *ω_e_* ranges from a few GHz to a few tens GHz for most of 1D, 2D and 3D PCs^30^,^31^, then the corresponding coupling strengths used in our calculations are ranged from a few MHz to a few hundreds MHz. The cutoff parameters Ω*_d_* ≈ 3.87*ω_e_* and Ω*_C_* = 0.1*ω_e_*; and the logarithmic divergence center *ω*_0_ = 1.04*ω_e_*.

In principle, the DOS for different PCs, denoted by 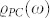, should be calculated by solving photon eigenfrequencies and eigenfunctions from the Maxwell's equations for different photonic crystal structures^2^,^23^,^24^. In the literature, different photonic DOS have been introduced for different PCs^9^,^10^,^11^,^12^,^14^,^25^,^26^,^27^,^28^,^29^. For examples, for 1D PCs, the corresponding DOS is given by 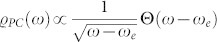, where Θ(*ω* − *ω_e_*) is the Heaviside step function and *ω_e_* is the frequency at the PBE. This DOS is also often used for isotropic 3D PCs, which could predict qualitatively correct behaviors of the non-Weisskopf-Winger decay and the photon-atom bound state in PCs^9^,^10^,^11^,^12^,^14^, but it may overestimate the spontaneous emission rate of atoms due to the absence of the singularity of DOS in reality. Thus, for 3D PCs, the DOS near the PBE may be modeled by an anisotropic DOS^9^,^10^,^11^: 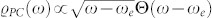, which could be further modified when the vectorial property of EM field is taken into account^25^,^26^. For 2D PCs with van Hove saddle point singularity^27^,^28^,^29^, the photonic DOS exhibits a logarithmic divergence near the PBE and can be approximated by 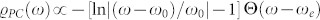, where *ω*_0_ is the center of the logarithmic peak. The spectral density *J*(*ω*) is microscopically defined as the multiplication of the DOS of the PCs with the photon tunneling amplitude *V*(*ω*) between the cavity and the PCs,
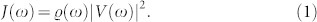
Assume that the cavity mode equally couples to all possible modes near the PBE of PCs, i.e. treating *V*(*ω*) as a constant, then the corresponding spectral densities *J*(*ω*) for different dimensional PCs are fully determined by the corresponding DOS, which are summarized in Table 1 and are also plotted in Fig. 1(a). We take the unit 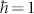 hereafter so that both the frequency *ω* and the spectral density *J*(*ω*) have the same unit as the energy.

Consider the cavity frequency *ω_c_* lies not too far away from the PBE. The dissipative photon dynamics is described by the cavity field propagating Green function *u*(*t*, *t*_0_) in photonic crystals through the relation 〈*a*(*t*)〉 = *u*(*t*, *t*_0_) 〈*a*(*t*_0_)〉, and is determined by the dissipative integro-differential equation (19). The general solution of Eq. (19) is given in Ref. 15,
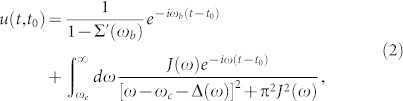
where 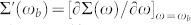 and Σ(*ω*) is the reservoir-induced cavity photon self-energy correction,
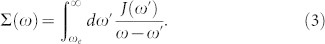
The explicit solution of cavity photon self-energy corrections for different spectral densities is also presented in Table 1. The frequency *ω_b_* in Eq. (2) is the localized photon mode frequency located inside the PBG (0 < *ω_b_* < *ω_e_*) but not too far away from the PBE, and it is determined by the pole condition: *ω_b_* − *ω_c_* − Δ(*ω_b_*) = 0, where 
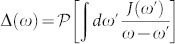
 is a principal-value integral.

Equation (2) provides a general solution of the non-Markovian dissipative photon dynamics for micro/nano cavities in various photonic crystal structures. It shows that the cavity photon dynamics in PCs always contains two parts^15^: a localized photon mode (the first term) plus a non-exponential photon damping (the second term). The localized photon mode is a long-lived non-Markovian effect (dissipationless), induced by the PBG structure in PCs. The corresponding frequency *ω_b_* lies always within the PBG. The non-exponential photon damping comes from the non-analyticity of the photon self-energy correction, which is determined by the DOS profile of the PCs. This non-exponential damping is a short-time non-Markovian memory effect, and it will become an exponential (Markov) decay in the photonic band (PB) region, as we will show later. The contributions of both the localized photon mode and the non-exponential photon damping strongly rely on the detuning *δ* = *ω_c_* − *ω_e_*.

In Fig. 1b, we plot the localized photon mode frequency as a function of the detuning *δ* for 1D, 2D and 3D PCs, respectively. The values of the localized mode frequencies for three different dimensional PCs are indeed very close to each other. However, a carefully check (see the inset in Fig. 1b) shows that for 3D PCs with an anisotropic DOS, the localized photon mode exists only when 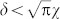, namely, the cavity frequency *ω_c_* must be tuned into the PBG or near the PBE. Here *χ* is a coupling strength between the cavity and the PCs (see Table 1). For 1D and 2D PCs, the localized photon mode exists for any location of the cavity frequency *ω_c_*. This is because for 1D and 2D PCs with the DOS given in Table 1, the self-energy corrections are negatively divergent when *ω* → *ω_e_* − 0^+^ so that the pole condition is always satisfied for the localized photon mode. However, this does not ensure that the localized photon mode in 1D and 2D PCs must have a significant contribution to the photon dynamics for the whole range of the cavity frequency. The importance of the localized photon mode is determined by the localized photon mode amplitude, 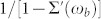 given in Eq. (2), which is plotted in Fig. 1c as a function of the detuning *δ* for all the three different dimensional PCs. It shows that for 3D PCs with an anisotropic DOS, the localized photon mode amplitude vanishes for 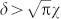, indicating a critical transition for the occurrence of the localized mode. For 1D and 2D PCs, the localized photon mode amplitude decays to almost zero when *ω_c_* is tuned into the PB region, as a crossover phenomenon. However, the overall effect of these localized photon modes on cavity photon dynamics has the very similar behavior for different PCs given in Table 1, as shown in Fig. 1.

**Figure 2:**
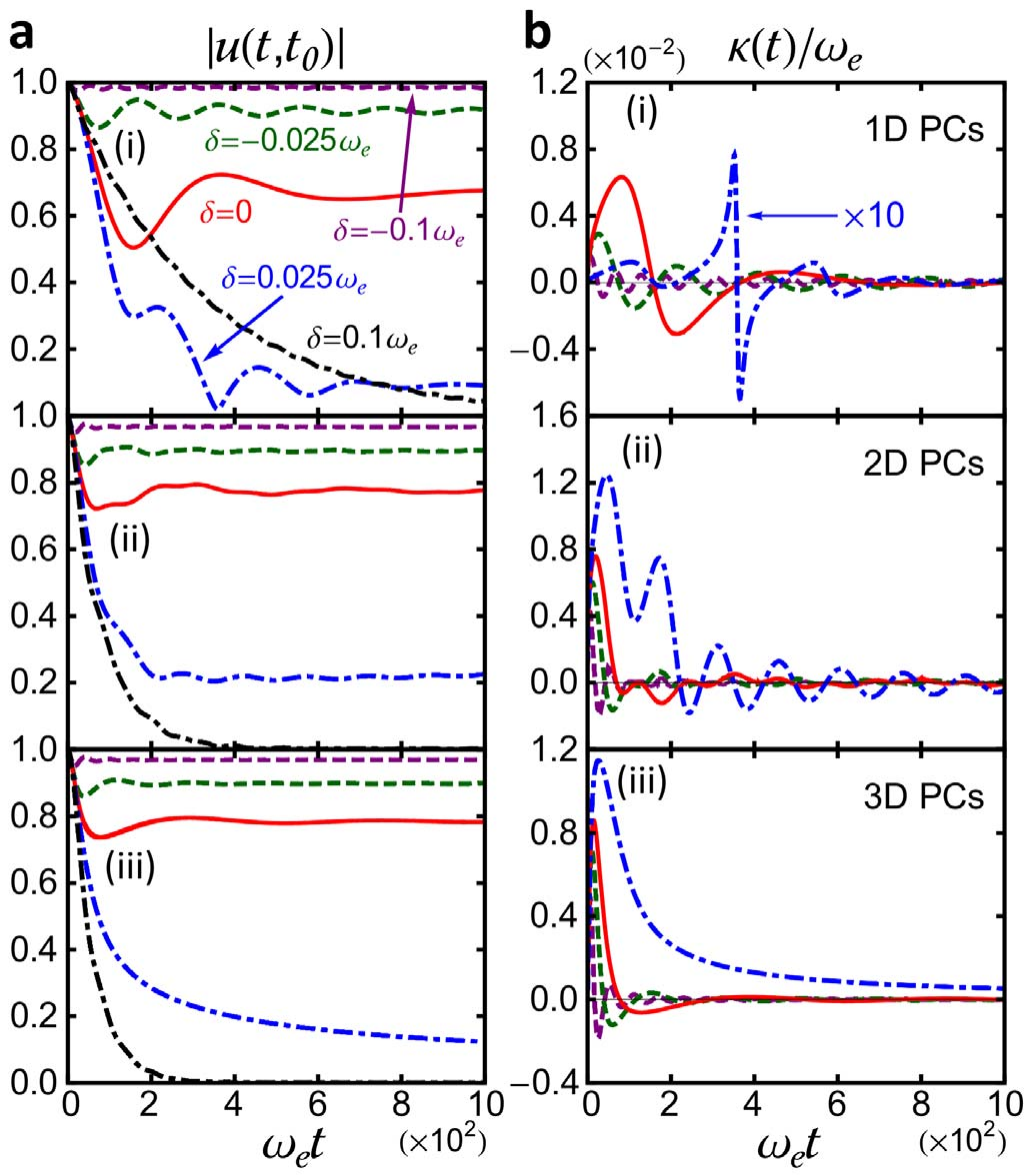
Dissipation and dissipationless photon dynamics. (a) Dissipation and dissiptionless photon dynamics in terms of the cavity field, 〈*a*(*t*)〉 = *u*(*t*, *t*_0_) 〈*a*(*t*_0_)〉, and (b) the corresponding decay rate *κ*(*t*), are plotted for (i) 1D PCs; (ii) 2D PCs and (iii) 3D PCs, with several different detuning *δ*. It shows how the dissipative cavity photons becomes dissipationless when the cavity frequency moves from the PB region into the PBG region for different dimensional PCs. For *δ* = 0.1*ω_e_*, the photon dynamics exhibits almost a perfect exponential decay, and by fitting the damping with an exponential function, the resulting damping rate is *κ* = 3.16 × 10^−3^*ω_e_*, 1.24 × 10^−2^*ω_e_* and 1.72 × 10^−2^*ω_e_* for 1D, 2D and 3D photonic crystals, respectively. Here *t*_0_ = 0, and other parameters are the same as given in Fig. 1.

The results presented in Fig. 1b–c provide indeed the full steady-state information of the cavity photon dynamics. This is because the non-exponential photon damping [i.e. the second term in Eq. (2)] will decay to zero so that only the localized photon mode contributes to the steady-state cavity photon field. In other words, the steady-state cavity field amplitude quantifies the contribution of the localized photon mode as a dissipationless effect, given by
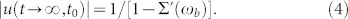
On the other hand, the non-exponential damping term in Eq. (2) characterizes the time-dependent cavity photon damping rate *κ*(*t*) in the intermediate dissipation time region through the relation^15^,^16^
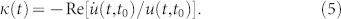
The detailed photon dynamics in terms of the cavity field amplitude |*u*(*t*, *t*_0_)| is provided in Fig. 2(a) for 1D, 2D and 3D PCs, respectively, with several different detuning *δ* taking from the PBG region to the PB region. The corresponding damping rate *κ*(*t*) is given in Fig. 2b, except for the case with *δ* = 0.1*ω_e_* which will be discussed later. The results show that the cavity dynamics changes dramatically when *ω_c_* crosses over from the PBG region to the PB region. Since the range of *u*(*t*, *t*_0_) is given by 1 ≥ |*u*(*t*, *t*_0_)| ≥ 0, we define the crossover region with the condition 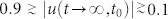 which corresponds to 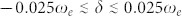, as shown in Fig. 1. The results in Fig. 2b show that the damping rate *κ*(*t*) will rapidly approach to zero within the PBG (*δ* < −0.025*ω_e_*) and in the vicinity of the PBE (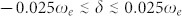). In other words, the photon damping rate will approach to zero after some time, due to the existence of the localized photon dynamics. In the PB region (*δ* > 0.025*ω_e_*) where either the localized mode vanishes (for 3D PCs) or becomes negligible (for 1D and 2D PCs), then the cavity photons undergo a full dissipation process, and can be approximately characterized by an exponential damping for 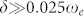, as shown by the black curves in Fig. 2a for *δ* = 0.1*ω_e_*. In this case, the damping rate *κ*(*t*) will oscillate rapidly in time when *u*(*t*, *t*_0_) decays to zero, see Eq. (5). This rapidly oscillating damping rate, originated from *u*(*t*, *t*_0_) approaching zero, has no physical consequence because the photon dissipation is almost completed after this point of time. Thus, alternatively we determine the damping rate *κ* by fitting the dashed-dot black curves in Fig. 2a with an exponential function ~exp(−*κt*). The resulting cavity photon damping rate for *δ* = 0.1*ω_e_* is given by 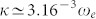, 1.24 × 10^−2^*ω_e_*, and 1.72 × 10^−2^*ω_e_* for 1D, 2D and 3D PCs, respectively. Experimentally, the typical photonic band edge frequency *ω_e_* ranges from a few GHz to a few tens GHz for most of 1D, 2D and 3D PCs fabricated in the microwave regime^30^,^31^, this corresponds to the damping rate *κ* ranging from a few tens MHz to hundreds MHz when the cavity frequency *ω_c_* lies inside the PB region.

Combining the localized photon mode together with the photon dissipation dynamics, as shown in Fig. 1 and Fig. 2, we can see that when the cavity mode is tuned away from the PBE with *δ* > 0.025*ω_e_*, the localized mode has a negligible effect on cavity photon dynamics for 1D and 2D PCs, and vanishes completely for 3D PCs. The cavity photon field decays almost exponentially for 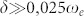, which is a Markov decay, as shown in Fig. 2a by the dashed-dot black curves with *δ* = 0.1*ω_e_*. In the crossover region 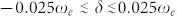, i.e. the cavity frequency is tuned into the vicinity of the PBE, the contribution of the localized mode to the photon dynamics increases rapidly, while the contribution of the nonexponential photon damping goes in an opposite way, namely the damping rate *κ*(*t*) is decreased to zero after some time (see Fig. 2b). When the cavity frequency lies inside the PBG (*δ* < −0.025*ω_e_*), the cavity field has almost no damping (the damping rate *κ*(*t*) quickly decays to zero), and the photon dynamics is dominated by the localized photon mode. Thus light can be confined in the defect of the photonic crystal, providing a high-Q micro/nano cavity. In fact, this nontrivial dissipative cavity photon dynamics reproduces the same result with regard to atomic population trapping and atom-photon bound states in the vicinity of the PBG where an atom is placed in photonic crystals^9^,^10^,^11^,^12^.

The importance of the above results is that the localized photon mode provides dissipationless cavity photon dynamics when the cavity frequency *ω_c_* lies inside the PBG or in the vicinity of the PBE. This dissipationless localized-mode contribution to the steady-state cavity photon field is universal for different dimensional PCs with different DOS given in Table 1, because it only relies on the presence of PBG. The localized photon mode decreases rather quickly but smoothly in the crossover region (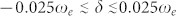), and becomes negligible for *δ* > 0.025*ω_e_* for 1D and 2D PCs, where the cavity photon dynamics becomes dissipative. For the 3D PCs, the cavity photon dynamics shows the same dissipationless behavior before reaching the critical point 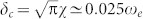, where the localized photon mode dominates the photon dynamics inside the PBG (*δ* < −0.025*ω_e_*) and decays rapidly near the PBE (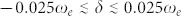). When *δ* > *δ_c_*, the localized photon mode vanishes for 3D PCs, and the cavity photon dynamics becomes fully dissipative. With these results, a general picture for dissipation and dissipationless photon dynamics in photonic crystals is provided.

### Thermal photon fluctuations

The above exact solution of dissipation and dissipationless cavity photon dynamics in photonic crystals can be used to describe thermal photon fluctuations through the fluctuated photon correlation function *v*(*t*, *t*), which is fully determined by the generalized non-equilibrium fluctuation-dissipation theorem^15^,
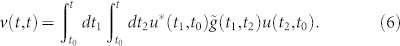
In Eq. (6), the two-time correlation function 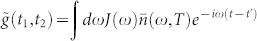 depicts photon fluctuations induced by the thermal photonic crystal, where 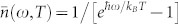 is the initial photon distribution in PCs at temperature *T*. When the cavity approaches the steady state, photon fluctuations are simply determined by the modified steady-state fluctuation-dissipation theorem, 
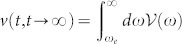
 with
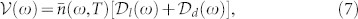
where 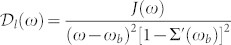 and 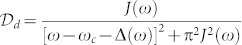. The first term is the localized photon mode contribution that modifies the conventional equilibrium fluctuation-dissipation theorem. This additional contribution is negligible when the cavity frequency is tuned into the PB region, i.e. *δ* > 0.025*ω_e_*, where *ω_b_* → *ω_e_* so that 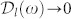 (see Fig. 1b–c). Consequently, the solution (7) is reduced to the standard equilibrium fluctuation-dissipation theorem: 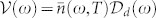. In the high temperature limit, it reproduces Einstein's fluctuation-dissipation relation.

**Figure 3:**
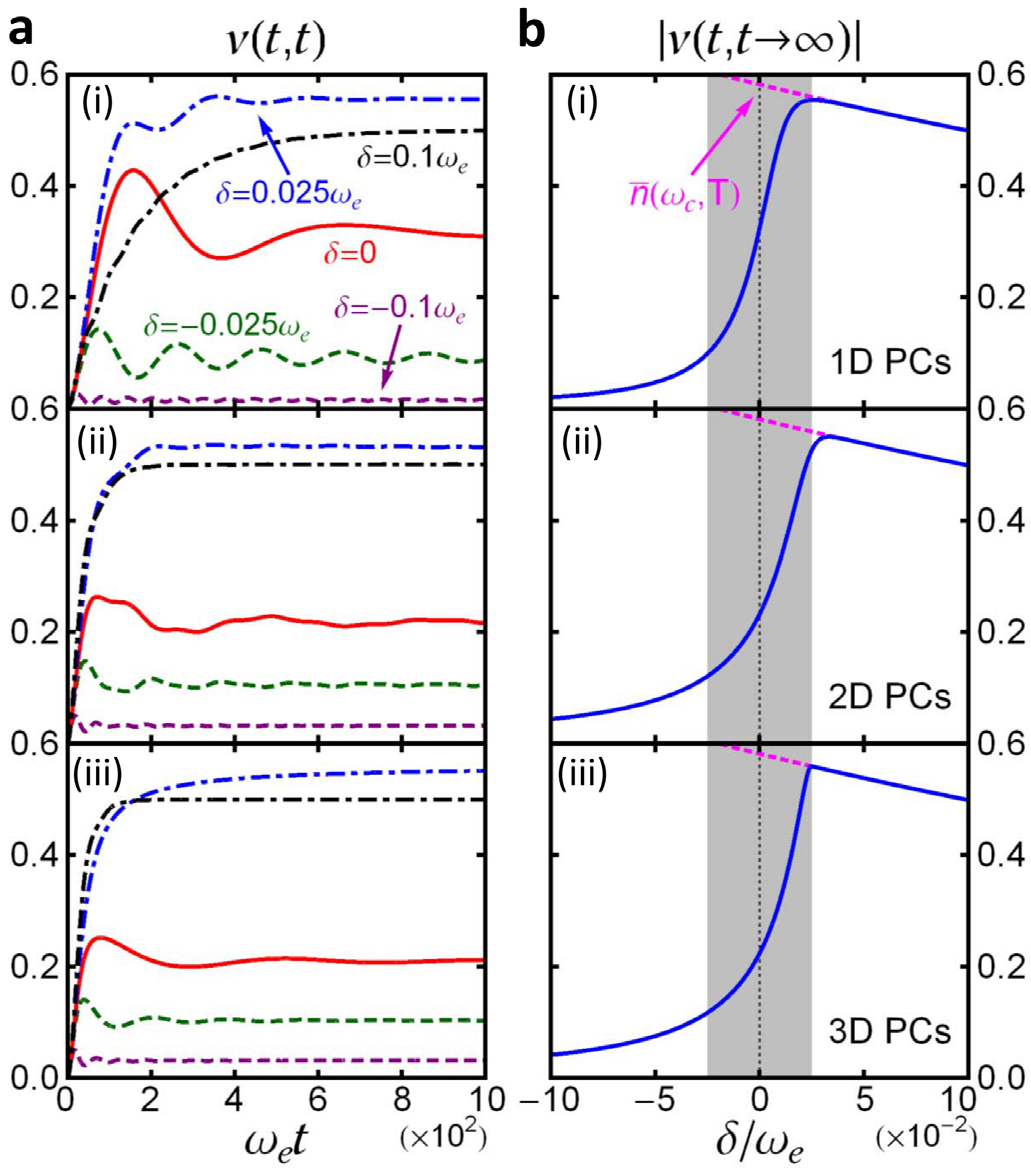
Fluctuated photon dynamics. (a) Time-evolution of cavity photon fluctuations, in terms of the photon correlation function *v*(*t*, *t*), are plotted for (i) 1D PCs, (ii) 2D PCs and (iii) 3D PCs. Different curves correspond to different detuning *δ* = *ω_c_* − *ω_e_*, as also shown in Fig. 2a, with the photonic crystal temperature 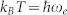; (b) The corresponding steady-state values of photon fluctuations, given by the solid-blue curve, as a function of the detuning. The pink-dashed curve is the result of the Bose-Einstein distribution 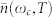.

Photon fluctuations are presented in Fig. 3a(i)–(iii) respectively for 1D, 2D and 3D PCs at a given temperature. It shows that photon fluctuations evolve in a similar way for different PCs. When *ω_c_* is tuned into the PB region (*δ* > 0.025*ω_e_*), photons continuously flow into the cavity from the photonic crystal until the cavity reaches its steady state. In this case, the cavity thermally equilibrates with the photonic crystal. When *ω_c_* lies inside the PBE region (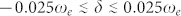), photons also flow into the cavity at the beginning, but some of photons are then transmitted back into the photonic crystal, due to the environment-induced memory effects and the effect induced by the localized photon mode. When *ω_c_* is tuned into the PBG region (*δ* < −0.025*ω_e_*), photons hardly flow from the photonic crystal into the cavity, because the localized photon mode dominates the cavity photon dynamics in the PBG region where the reservoir-induced dissipation is negligible, even though the thermal energy, 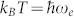, is much larger than the detuning energy. Thus, thermal fluctuations are suppressed significantly by the photon localization in the PBG region and also near the PBE.

The physical picture of the above photon fluctuations can be seen clearly by connecting the photon correlations with the measurable average cavity photon number (i.e. field intensity)^16^,^18^,^32^: *n*(*t*) = 〈*a*^†^(*t*)*a*(*t*)〉 = |*u*(*t*, *t*_0_)|^2^ *n*(*t*_0_) + *v*(*t*, *t*), where *n*(*t*_0_) is the initial cavity intensity. It shows that the fluctuated photon correlation function *v*(*t*, *t*) is just the thermal-fluctuation-induced average photon number in the cavity. In Fig. 3b, we present the steady-state photon fluctuations at a given temperature, see the solid-blue curve. The dashed-pink curve is the thermal photon distribution in PCs at the same temperature: 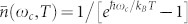. It shows that when *δ* > 0.025*ω_e_*, the thermal-fluctuation-induced steady-state average photon number in the cavity is identical to the thermal photon distribution: 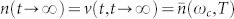. In this regime, the initial cavity photons are totally lost into PCs as *u*(*t*, *t*_0_) → 0 in the steady-state limit, as a consequence of the Weisskopf-Winger decay. The steady-state cavity photons all come from thermal fluctuations of the reservoir. As a result, the cavity photons approach equilibrium with the reservoir and obey Bose-Einstein distribution.

However, near the PBE (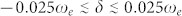), the thermal-fluctuation-induced photon number deviates significantly from the thermal photon distribution, as shown in Fig. 3b. In other words, Bose-Einstein distribution no longer works for cavity photons in the PBE region, due to the localized photon effect. When the cavity frequency *ω_c_* lies inside the PBG (*δ* < −0.025*ω_e_*), the thermal fluctuations approach to zero, and thus the cavity is fully determined by the localized photons, even though the cavity mode equally couples to all possible modes in PCs and the thermal energy is much larger than the detuning, 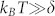. Consequently, Bose-Einstein distribution is broken down. This provides a nontrivial phenomenon of the thermal fluctuation dynamics from equilibrium to nonequilibrium steady states. Here nonequilibrium steady states are defined by these steady states which can memorize the initial state information of the system so that the equilibrium hypothesis cannot be satisfied. Such steady states will be given explicitly in the next subsections.

It is recently shown that non-Markovian dynamics in open systems can induce a critical transition from equilibrium to nonequilibrium steady states, when a localized mode occurs^15^,^22^,^33^. Such a critical transition appears for the 3D PCs with an anisotropic DOS, where there is a critical detuning for the occurrence of localized photon mode, 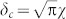. However, for the 1D and 2D PCs, our result shows that there is no such a critical transition due to the fact that the localized photon mode always exists. The photon fluctuations then undergoes a crossover from equilibrium to nonequilibrium steady states through the change of the detuning *δ*. No matter it is a crossover or a critical transition, the results in Fig. 3b shows that thermal photon fluctuations cannot make the cavities embedded in PCs to approach equilibrium with the PCs when the cavity frequency lies inside the PBG or near the PBE. This is a general feature for cavity photons in PCs.

### Breakdown of Bose-Einstein distribution through the time-evolution of photonic Fock states

Based on the exact cavity photon dissipation and fluctuation dynamics in PCs, we shall explicitly examine the breakdown of Bose-Einstein distribution through the evolution of cavity photonic states, and also the crossover from equilibrium to nonequilibrium steady states. This must be done by solving the exact master equation (15) given in the Methods. To be more specific, we consider first the cavity to be initially in a Fock state with an arbitrary photon number *n*_0_, i.e. *ρ*(*t*_0_) = |*n*_0_〉 〈*n*_0_|, which may be prepared experimentally through the real-time quantum feedback control^34^. By solving the master equation (15), the cavity state at arbitrary time *t* is given by
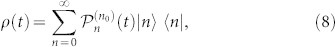

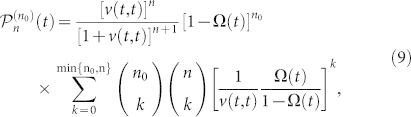
where 
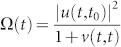
. This result shows that an initial photon Fock state will evolve into a mixed state of different Fock states, and the probability in each Fock state |*n*〉 is 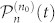.

**Figure 4:**
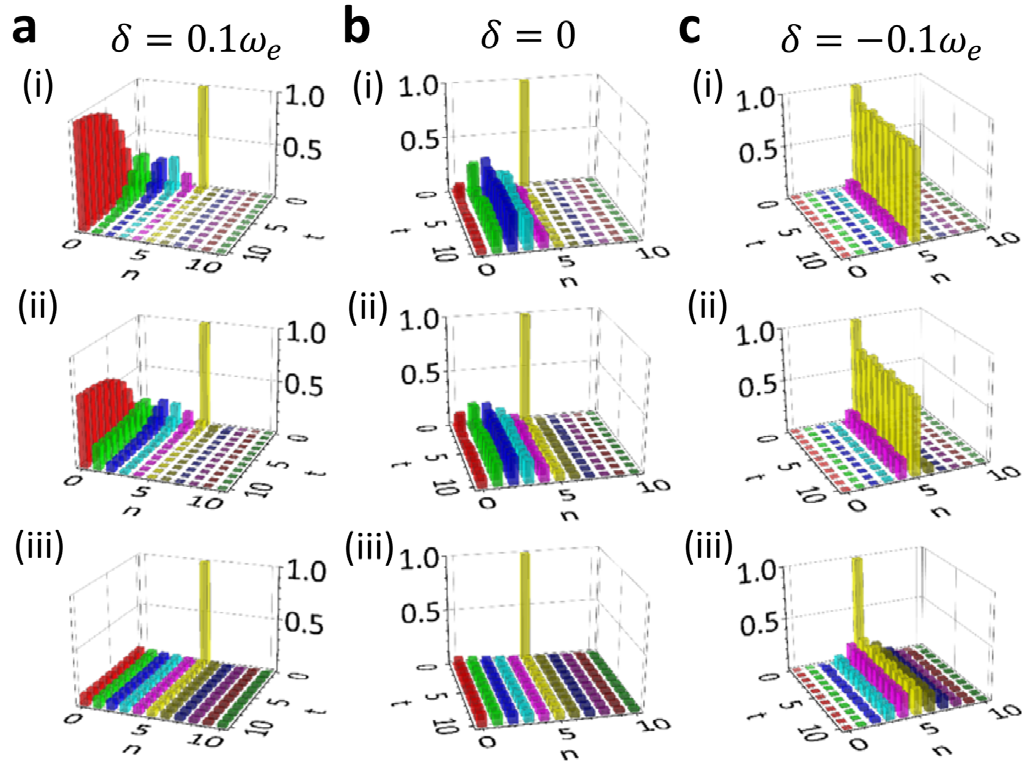
Time-evolution of Fock states. The time-evolution of the probability distribution, 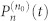 with different detuning *δ*: (a) *δ* = −0.1*ω_e_*; (b) *δ* = 0; and (c) *δ* = 0.1*ω_e_*, for photonic crystals at different temperature *T*: (i) 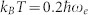; (ii) 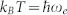; and (iii) 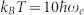. The cavity is initially prepared in the Fock state |*n*_0_ = 5〉. The time *t* is scaled by 10^2^*ω_e_*.

**Figure 5:**
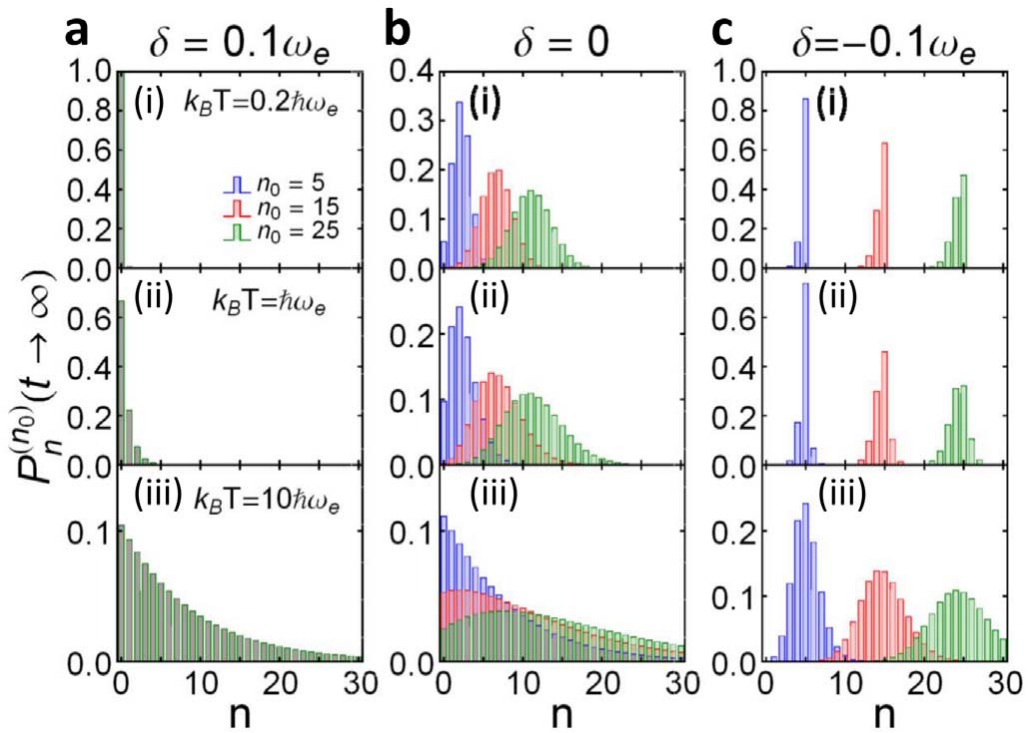
Steady-state photon distributions I. The steady-state cavity photon distribution, 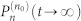 for different initial Fock states |*n*_0_〉, *n*_0_ = 5, 15 and 25 (in terms of different colors); with different detuning *δ*: (a) *δ* = 0.1*ω_e_*, (b) *δ* = 0, and (c) *δ* = −0.1*ω_e_*; and different temperatures *T* of the photonic crystals: (i) 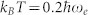, (ii) 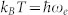, and (iii) 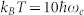, as given in the figure.

As we have shown, the breakdown of Bose-Einstein distribution relies on the dissipationless photon dynamics. And different dimensional PCs show almost the same dissipationless photon dynamics, determined by the localized photon mode due to the existence of the PBG. To be specific, we will present in the following the numerical solution of the state evolution for 1D PCs. The 2D and 3D PCs must provide a similar solution, based on the universal dissipation and fluctuation photon dynamics provided by Eqs. (2) and (6), and also the explicit numerical results presented in Fig. 2 and Fig. 3. The time-evolution of the cavity photon distribution 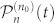 for the initial state |*n*_0_ = 5〉 is given in Fig. 4. The steady-state limit, 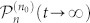, is shown in Fig. 5, where several different initial states have been considered to demonstrate the initial-state dependence of the steady photon states.

The results show that if we tune *ω_c_* into the PB region (e.g. *δ* = 0.1*ω_e_*), then *u*(*t*, *t*_0_) will gradually decay to zero. This indicates that photons in the cavity will gradually be damped into the photonic crystal, and photons in the photonic crystal are transferred into the cavity through thermal fluctuations. In this case, the contribution of the localized photon mode is negligible. The cavity state ultimately reaches thermal equilibrium with the photonic crystal, and Bose-Einstein statistical distribution is produced,
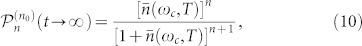
where the initial state information is completely washed out, and the cavity photon steady state is independent of the initial states, as shown in Fig. 4a and Fig. 5a.

Near the PBE (e.g. *δ* = 0), the cavity still loses photons into the photonic crystal and gains photons from the photonic crystal through thermal fluctuations. At a low temperature (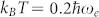), the photon loss and photon gain make the cavity become a mixed state of several Fock states |*n*〉 only for *n* < *n*_0_, and mainly distributed around *n* = *n*_0_/2, see Fig. 4b–(i) and also the steady-state limit by Fig. 5b–(i) where the initial state dependence is manifested. The photon distribution deviates significantly from the standard Bose-Einstein distribution. At a relatively high temperature (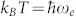), the cavity state also becomes a mixed state of several Fock states |*n*〉, distributed mainly among *n* ≤ *n*_0_/2, but the distribution is broader, see Fig. 4b–(ii) and also the steady-state limit in Fig. 5b–(ii). In this case, the initial-state dependence of the steady state is still shown up and the derivation of the photon distribution from the standard Bose-Einstein distribution is obvious. As the temperature becomes rather high (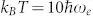), the time-evolution of the cavity state behaves quite differently because thermal fluctuations play a more important role now, thus the structure of the initial state is quickly destroyed, as shown in Fig. 4b–(iii). However, the cavity does not really approach thermal equilibrium with the photonic crystal, because 
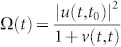
 is small but not negligible so that the distribution given by Eq. (9) is still different from the thermal state distribution. The cavity steady states for different initial states do not differ from each other as much as these in the low temperature cases, but the initial-state dependence is still observable, as shown in Fig. 5b–(iii).

When the cavity frequency *ω_c_* lies inside the PBG, e.g. *δ* = −0.1*ω_e_* given in Fig. 4c and Fig. 5c, the steady-state cavity photon distribution strongly depends on the initial state. In particular, if the photonic crystal temperature is not too high (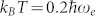 and 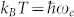), the cavity remains in its initial Fock state as a photon localization state. It only has a small chance to decay to the Fock state |*n*〉 with *n* < *n*_0_ [see Fig. 4c(i) with 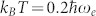], and an even smaller probability to be in the Fock state |*n*〉 with *n* > *n*_0_ [see Fig. 4c(ii) when *k_B_T* increases to 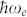]. The steady-state cavity photons are distributed mainly in the regime *n* < *n*_0_ with the maximum peak at *n* = *n*_0_, see Fig. 5c(i)–(ii). However, at a higher temperature 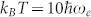, the thermal fluctuation becomes strong such that some of the initial state information will be lost and the cavity evolves into a mixed state covering several Fock states around the initial one |*n*_0_〉 [see Fig. 4c(iii)]. The steady-state cavity photons are distributed more broadly, but are still centered around the initial photon number *n*_0_, as shown in Fig. 5c(iii). This is because the localized photon mode dominates the photon dynamics over thermal photon fluctuations. The overall initial-state dependence of the steady states shown in Fig. 5b–c indicates that the equilibrium hypothesis in statistical mechanics is no longer obeyed and Bose-Einstein statistical distribution is completely broken down, even though the initial thermal energy of the photonic crystals is larger or much larger than the detuning energy.

### Breakdown of Bose-Einstein distribution through the time-evolution of coherent states

Because Fock sates are highly non-classical and may not be easy to prepare in experiments, here we examine the case the cavity is initially in a coherent state |*α*_0_〉. By solving the master equation (15), the cavity state at an arbitrary later time *t* is given by
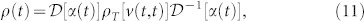
where 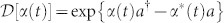 is a displacement operator with *α*(*t*) = *u*(*t*, *t*_0_)*α*_0_, and
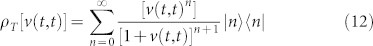
is a thermal-like state with average particle number *v*(*t*, *t*). Equation (11) shows that the initial cavity state will evolve into a displaced thermal-like state^35^,^36^ which is the mixture of displaced number states 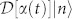
37. In the photon number representation, Eq. (11) can be written as
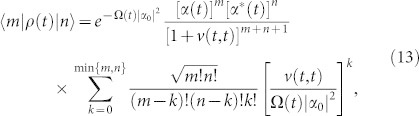
where the probability of the cavity containing *n* photons (i.e. cavity photon distribution) is given by the diagonal terms of Eq. (13).

**Figure 6:**
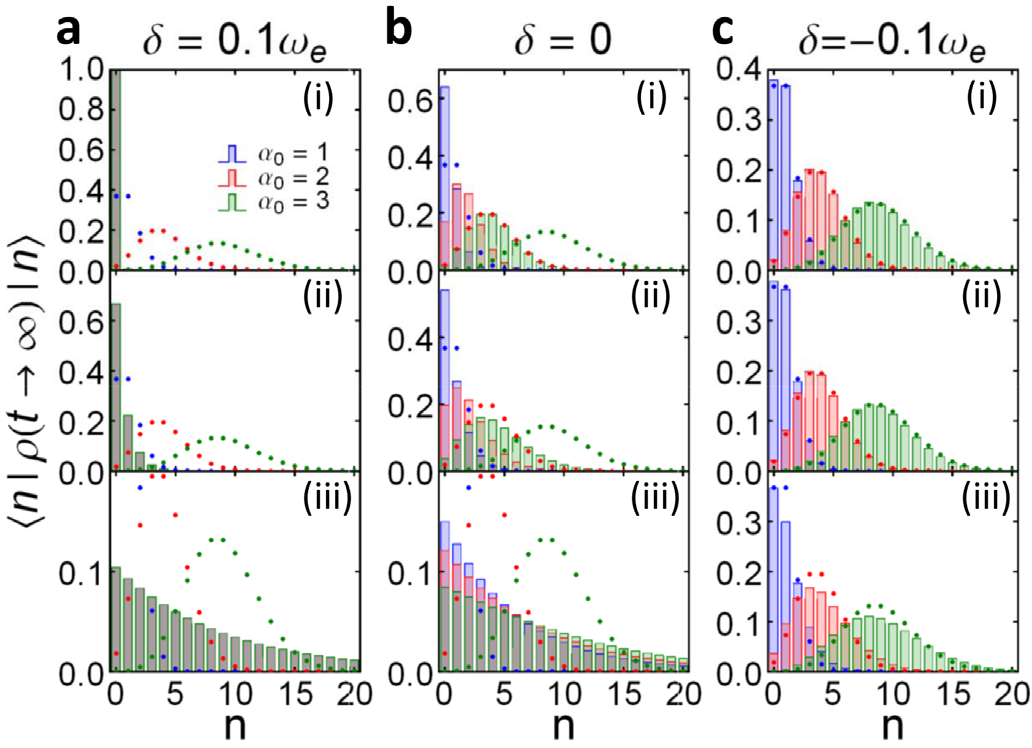
Steady-state photon distributions II. The steady-state cavity photon distribution (colored bars), 〈*n*| *ρ* (*t* → ∞) |*n*〉 for different initial coherent states |*α*_0_〉 (shown by the dotted curves with different colors); with different detuning *δ*: (a) *δ* = 0.1*ω_e_*, (b) *δ* = 0, and (c) *δ* = −0.1*ω_e_* at different temperatures of photonic crystals: (i) 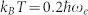, (ii) 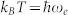, and (iii) 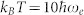.

The cavity photon distribution in the steady-state limit is presented in Fig. 6. It shows again that when the cavity frequency *ω_c_* lies inside the PB region (*δ* = 0.1*ω_e_*), the cavity state (11) will evolve into the thermal state 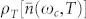, which indicates that the cavity photon number obeys Bose-Einstein distribution, see Fig. 6a. If the cavity mode is tuned at the PBE (*δ* = 0), the cavity steady state will depend on its initial state, as shown in Fig. 6b. For low temperature (e.g. 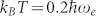), the thermal fluctuation is suppressed (*v*(*t*, *t*) → 0), and the cavity will evolve into the coherent state |*α*(*t*)〉, see Eq. (11). In this case, the cavity photon distribution in the steady state is Poissonian-like, with average photon number 〈*n*〉 |*_t_*_ → ∞_ = |*α*(*t*)|^2^ ∝ |*α*_0_|^2^, see Fig. 6b–(i). As the temperature goes higher (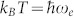), the cavity photon distribution is deviated away from the Poisson distribution due to thermal fluctuations, see Fig. 6b–(ii). At high temperature (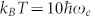), since thermal fluctuation plays a more important role, the initial-state dependence is not as significant as that in the low temperature, and the cavity photon distribution is not Poissonian-like, but still deviates from Bose-Einstein distribution, see Fig. 6b–(iii). When the cavity frequency lies inside the PBG (*δ* = −0.1*ω_e_*), the cavity steady state strongly depends on its initial state. The photon distribution in the cavity remains almost the same as in its initial state when the temperature is not very high (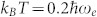 and 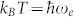), see the first two graphs in Fig. 6c. The initial-state dependence of the photon distribution is still strong when the temperature gets higher (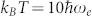), see Fig. 6c–(iii). The photon distribution in this case is slightly broadened from the initial states due to thermal fluctuations at high temperature, but still centered around *n* ≈ |*α*_0_|^2^, as a manifestation of photon localization. These results obtained for different initial coherent states at different temperature behave indeed in the same way as the results obtained for different initial Fock states under the same temperature.

## Discussion

The breakdown of Bose-Einstein distribution in PCs, explored in details through the time-evolution of various cavity photon states for 1D PCs, is also valid for 2D and 3D PCs with different DOS listed in Table 1. This is because the time evolution of the cavity photon states, solved analytically by Eqs. (8) and (11), is fully determined by the photon dissipation and fluctuation dynamics of Eqs. (2) and (6). The steady-state behaviors of Eqs. (2) and (6) for different PCs are almost the same, as shown in Fig. 2 and Fig. 3. There is only a slight difference on the photon dissipation and fluctuation dynamics between 1D and 2D PCs with 3D PCs. For 1D and 2D PCs, varying the detuning leads to a crossover from equilibrium to nonequilibrium steady states for the photon dynamics, while for 3D PCs with an anisotropic DOS, it gives a critical transition, rather than a crossover, due to the existence of a critical detuning for the existence of the localized mode, see explicitly the results presented in Fig. 1 to Fig. 3. Such a critical transition is also found recently in other open systems^22^,^33^ but not for the crossover phenomenon. Thus, photon dissipation and fluctuation dynamics investigated in this work reveal a new nontrivial property, i.e. a crossover phenomenon for photon dynamics in low-dimensional photonic crystals.

In conclusion, we show that when the cavity frequency lies inside the PBG or near the PBE in photonic crystals, Bose-Einstein statistical distribution is broken down for cavity photons. This conclusion is generally valid for various photonic band gap structures in PCs. For the 1D and 2D PCs, the breakdown of Bose-Einstein distribution leads to a crossover from equilibrium to nonequilibrium cavity steady states, while for 3D PCs with an anisotropic DOS, the breakdown of Bose-Einstein distribution corresponds to a critical transition rather than a crossover. No matter whether it is a crossover or a critical transition, the breakdown of Bose-Einstein distribution is a consequence of localization photons due to the presence of PBG structures in PCs. Therefore the conclusion is also valid for other nanomaterials with band gap structures. It could provide a hitherto unexplored challenge on photon statistics. Furthermore, this non-trivial photon dynamics can be examined via quantum non-demolition measurement^38^,^39^, by sending sequences of circular Rydberg atoms through the photonic crystal micro/nano cavity, which carry information without destroying the cavity photon state. In particular, the cavity photon state can be measured using an experimental setup similar to that given in Ref. 38. Such experiments could be done with microcavities in low-dimensional photonic crystals in the microwave regime.

## Methods

To investigate photon dynamics of micro/nano cavities which are coupled each other through waveguides embedded in PCs at finite temperature, we treat both the PCs and waveguides as reservoirs of the cavities. Thus the entire system of the micro/nano cavities (defects) embedded in photonic crystals can be described by the Fano-Anderson model (a model of impurity electrons coupled with continuous states introduced by Anderson^40^ in solid-state physics, and discrete states embedded in a continuum proposed by Fano^41^ in atomic spectra). The corresponding Fano-Anderson Hamiltonian is given by Refs.^16^, ^40^,^41^,^42^,^43^:
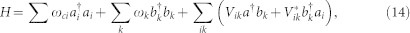
where 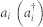 is the annihilation (creation) operator of the micro/nano cavity modes (defects), and 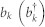 the annihilation (creation) operator of the photonic modes of photonic crystals (continuum). The coefficients *V_ik_* are tunneling amplitudes of photons between the micro/nano cavities and the photonic crystals. The Hamiltonian (14) has also the same form as the one for cavity photon loss in open space^20^, by replacing the photonic crystals with an open space.

Consider initially the photonic crystals are in equilibrium state. Integrating out completely the reservoir degrees of freedom of photonic crystals via the influence functional^44^,^45^ in the coherent state representation^46^, arbitrary photon states for micro/nano cavities in PCs are then governed by the following exact master equation^15^,^16^,^17^,^18^,^32^,^43^,^47^,^48^
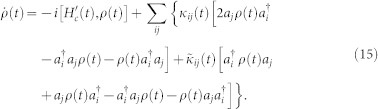
Here *ρ* (*t*) is the reduced density matrix for cavity states, 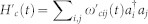 is the renormalized Hamiltonian of cavities with the environment-modified cavity frequencies 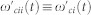 and the environment-induced couplings between different cavities 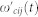, after the environmental degrees of freedom are completely integrated out. The coefficients *κ_ij_* (*t*) and 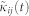 characterize photon dissipations and fluctuations in PCs at finite temperature. The renormalized frequency, 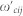, and the time-dependent dissipation and fluctuation coefficients, *κ_ij_* (*t*) and 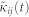, can be exactly and non-perturbatively determined by the dissipation-fluctuation Dyson equation, as shown explicitly in our early works^16^,^17^, and will also be given later.

It might be worth pointing out that the first master equation derived from the original Feynman-Vernon influence functional^44^ was obtained by Caldeira and Leggett for the Brownian motion with a high temperature environment^45^ which is also called the Caldeira-Leggett master equation in the literature. For the Brownian motion, the system-reservoir coupling Hamiltonian is given by 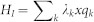, where *x* and *q_k_* are the positions of the principal harmonic oscillator (as the Brownian particle) and all other harmonic oscillators in the reservoir, respectively. In terms of the second quantization, this system-reservoir coupling Hamiltonian can be rewritten as 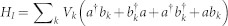 which shows that the amplitudes for particle tunneling processes between the system and the reservoir (the first two terms) is equal to the amplitudes of particle pair production and annihilation processes (the last two terms), both are given by *V_k_*. However, in quantum optics, it is well-known that the photon tunneling amplitudes cannot be the same as the amplitudes of two-photon pair production and annihilation processes. As a result, the Caldeira-Leggett model is not applicable to photonics.

To make the cavity photon dynamics in thermal photonic crystals more specific, we consider a single-mode micro/nano cavity in photonic crystals. The photon dissipation and fluctuations, characterized by the dissipation and fluctuation coefficients *κ* (*t*) and 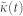 (all the sub-indices (*i*, *j*) in Eq. (15) can be dropped now), are determined non-perturbatively and exactly by the nonequilibrium Green functions^49^,^50^ through the relations^15^,^16^,^17^,^18^:
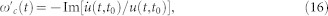

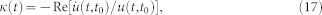

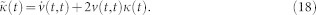
Here *u* (*t*, *t*_0_) is the cavity photon field propagating Green's function describing the photon field relaxation, and *v* (*t*, *t*) characterizes the reservoir-induced photon thermal fluctuations. These two Green functions, *u* (*t*, *t*_0_) and *v* (*t*, *t*), are determined by the following integrodifferential dissipation equation and the nonequilibrium fluctuation-dissipation theorem, respectively^15^,^16^,
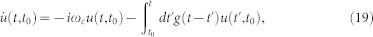

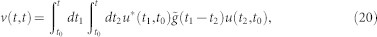
where *ω_c_* is the original cavity frequency. The integral kernels in Eqs. (19)–(20) characterize all the back-actions between the cavity and photonic crystals, and can be determined uniquely by the spectral density *J* (*ε*) of photonic crystals through the relations: 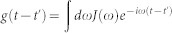, and 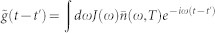, where 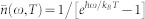 is the initial photon distribution in PCs at temperature *T*. The spectral density *J*(*ω*) is microscopically defined as a multiplication of the density of states (DOS) 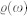 of PCs with the photon scattering amplitudes *V_k_* between the cavity and PCs,
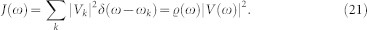
In the second equality we have taken the continuous photonic modes of PCs so that *V_k_* → *V*(*ω*), and the index *i* of *V_ik_* in Eq. (14) has also been dropped for single-mode cavity. For arbitrary spectral density *J*(*ω*), the general solution of Eq. (19) can be obtained exactly^15^, which is given by Eq. (2). By solving Eqs. (16)–(18) and (15) through Eqs. (19)–(20), the complete solution of photon dissipation and fluctuations can be obtained, as we presented in details in the paper.

## Author Contributions

W.M.Z. proposed the ideas, interpreted the physics and wrote the main manuscript, P.Y.L. performed the theoretical calculations and prepared all the figures, and H.N.X. checked the results and participated the discussions. All authors reviewed the manuscript.

## Figures and Tables

**Table 1:** Characters of different photonic crystal structures. For different DOS of different dimensional PCs, the corresponding different spectral densities *J*(*ω*) are listed and the reservoir-induced photon self-energy corrections Σ(*ω*) are calculated, which completely determine the dissipative photon dynamics in PCs. The parameters *C*, *η* and *χ* are coupling strengths (with a proper rescaling) between the cavity and PCs for 1D, 2D and 3D PCs, respectively. A smooth high-frequency cutoff Ω*_C_* is introduced for avoiding the divergence of DOS in 3D PCs, and a sharp high-frequency cutoff at Ω*_d_* is set up for maintaining the positivity of DOS in 2D PCs. Here Li_2_ (*x*) is a dilogarithm function and erfc (*x*) is a complementary error function

Photonic Crystals (PCs)	Spectral density *J*(*ω*) for different DOS	Reservoir-induced self-energy correction Σ(*ω*)
1D	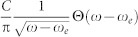	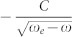
2D	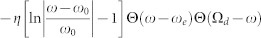	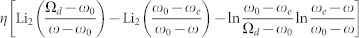
3D